# Motor signature of autism spectrum disorder in adults without intellectual impairment

**DOI:** 10.1038/s41598-022-10760-5

**Published:** 2022-05-10

**Authors:** An Bin Cho, Karen Otte, Irina Baskow, Felicitas Ehlen, Tolou Maslahati, Sebastian Mansow-Model, Tanja Schmitz-Hübsch, Behnoush Behnia, Stefan Roepke

**Affiliations:** 1 Department of Psychiatry and Psychotherapy, Charité - Universitätsmedizin Berlin, Campus Benjamin Franklin, Hindenburgdamm 30, 12203 Berlin, Germany; 2 Motognosis GmbH, Schönhauser Allee 177, 10119 Berlin, Germany; 3 Experimental and Clinical Research Center, Charité – Universitätsmedizin Berlin, corporate member of Freie Universität Berlin and Humboldt-Universität zu Berlin, Lindenberger Weg 80, 13125 Berlin, Germany; 4 Experimental and Clinical Research Center, a cooperation between the Max-Delbrück-Center for Molecular Medicine in the Helmholtz Association and the Charité - Universitätsmedizin Berlin, Charitéplatz 1, 10117 Berlin, Germany; 5 Max-Delbrück-Center for Molecular Medicine, Helmholtz Association (MDC), Robert-Rössle-Straße 10, 13125 Berlin, Germany; 6 Department of Psychiatry and Psychotherapy, Jüdisches Krankenhaus Berlin, Heinz-Galinski-Str. 1, 13347 Berlin, Germany; 7 NeuroCure Clinical Research Center, Charité – Universitätsmedizin Berlin, corporate member of Freie Universität Berlin and Humboldt Universität Zu Berlin, Charitéplatz 1, 10117 Berlin, Germany

**Keywords:** Psychology, Biomarkers, Diseases, Medical research, Signs and symptoms

## Abstract

Motor signs such as dyspraxia and abnormal gait are characteristic features of autism spectrum disorder (ASD). However, motor behavior in adults with ASD has scarcely been quantitatively characterized. In this pilot study, we aim to quantitatively examine motor signature of adults with ASD without intellectual impairment using marker-less visual-perceptive motion capture. 82 individuals (37 ASD and 45 healthy controls, HC) with an IQ > 85 and aged 18 to 65 years performed nine movement tasks and were filmed by a 3D-infrared camera. Anatomical models were quantified via custom-made software and resulting kinematic parameters were compared between individuals with ASD and HCs. Furthermore, the association between specific motor behaviour and severity of autistic symptoms (Autism Diagnostic Observation Schedule 2, Autism Spectrum Quotient) was explored. Adults with ASD showed a greater mediolateral deviation while walking, greater sway during normal, tandem and single leg stance, a reduced walking speed and cadence, a greater arrhythmicity during jumping jack tasks and an impaired manual dexterity during finger tapping tasks (*p* < 0.05 and |D|> 0.48) compared to HC. Furthermore, in the ASD group, some of these parameters correlated moderately to severity of ASD symptoms. Adults with ASD seem to display a specific motor signature in this disorder affecting movement timing and aspects of balance. The data appear to reinforce knowledge about motor signs reported in children and adolescents with ASD. Also, quantitative motor assessment via visual-perceptive computing may be a feasible instrument to detect subtle motor signs in ASD and perhaps suitable in the diagnosis of ASD in the future.

## Introduction

Besides core symptoms of autism spectrum disorder (ASD), such as difficulties in social communication and interaction as well as restricted, repetitive patterns of behavior, interests, or activities, specific motor signs such as dyspraxia have been described in children and adolescents with ASD^1,2^. However, there is scarce information available on motor functions in adults with ASD^3^.

Specific motor signs in ASD were described as early as 1943 by Kanner, who observed motor “clumsiness” in children with ASD^4^. Since the neuroanatomical description of hypoplasia of the cerebellar vermal lobules of persons with ASD in 1988, there have been numerous endeavors to quantify motor functions^5^. A meta-analysis found that 21–100% of children and adolescents with ASD display pronounced changes in motor behaviours^2^. Evidence of impairments in inter-hemispheric coordination (corpus callosum) and neurological soft signs such as dysdiadochokinesia in children with ASD without intellectual impairment point towards a general distinction of motor functions^6–9^. Motor function distinction in children with ASD was associated with impairments in social, communicative, and behavioral skills, and thus, with the severity of ASD in general^2^.

Motor skills in children aged 3–16 are usually assessed using clinical assessment batteries, e.g. the Movement Assessment Battery for Children (MABC-2)^10^. MABC-2 comprises of performance tasks and a checklist. During the performance tasks, children complete a series of fine and gross motor tasks examining the domains “manual dexterity”, “balance”, and “aiming and catching”. Using a checklist, a parent or a caregiver evaluates the child´s motor functions on a 30-item scale. Perin and colleagues have carried out the Physiological Profile Assessment (PPA) in children with ASD and healthy controls, assessing the domains vision, peripheral sensation, lower limb muscle strength, simple reaction time and balance, and reinforcing previous findings on postural instability in children with ASD, among others^11^.

In recent years, there has been considerable development in quantitative assessment methods for different motor functions, e.g. pressure-sensitive walkways (e.g. GAITRite Walkway®), wearable motion sensors (e.g. Mobility Lab) or full-body motion capture using video-based systems (Vicon Motion Systems®, Motognosis Labs)^12–16^. These new technologies have been used to quantitatively assess motor functions in children with ASD, e.g. gait features using GAITRite Walkway® and other 3D-motion capture systems^17–21^ and postural control via stance tasks using force plates^22^. However, the preliminary data on motor function in persons with ASD is mostly based on children and adolescents.

“Clumsiness” observed in childhood ASD seems to prevail as seen in a 10-year follow-up study^23^. Also, the extent of motor signs in children with ASD seems to be positively associated with the severity of ASD after reaching adulthood^24^. In a study, in which ASD diagnosis was assessed via self-report in adults, clumsiness and other symptoms of dyspraxia were reported almost seven times as frequently as in healthy controls^3^. In the same study, dyspraxia in the general adult population was associated with greater autistic traits. In recent years, there have been some efforts to assess motor skills in adults with ASD. In a meta-analysis by Lum and colleagues, 3 out of 18 studies quantitatively assessed gait characteristics such as walking speed and gait cycle time specifically in adults with ASD, one of which only included four participants^16^. For example, gait analysis using GAITRite pressure sensitive walkway found reduced walking speed and decreased postural stability in young adults with ASD^25^. Reduced walking speed in adults with ASD was also seen in a study by Armitano and colleagues using a pressure-sensitive surface walkway (Zeno Walkway)^26^. Still, a quantitative, systemic assessment of various motor features in persons with ASD is still lacking.

However, information on motor skills is important for the diagnostic process. Especially adult individuals with ASD without intellectual impairment are either diagnosed very late or not at all, compared to individuals with ASD with intellectual impairment^27^. Often, the diagnosis of ASD is made as an incidental finding when adults with ASD without intellectual impairment seek treatment for a psychiatric comorbidity, e.g., depression^28^.

Nonetheless, the impact of motor signs in adults with ASD on the level of impairment and participation are yet to be determined. A more precise description of the motor signature of adult ASD will help to close this gap.

In this pilot study, we aim to assess the use of a clinically applicable assessment of various motor functions in adults with ASD without intellectual impairment. We derived a quantitative description of motor behavior from a short motor testing protocol using visual-perceptive computing. This protocol has previously been technically validated in healthy subjects^15^ and the method has been used in several patient groups, such as multiple sclerosis^29^, with high usability. The motor signature of ASD was defined by comparison to healthy controls (HC) and by investigating the relationship between motor parameters and severity of ASD syndrome. We hypothesize that adults with ASD without intellectual impairment would differ motor functions compared to healthy controls. Following findings in children with ASD, we hypothesize a reduced movement speed and reduced fine motor skills in adults with ASD without intellectual impairment. We also hypothesize an association between distinctive motor features of ASD and severity of autistic symptoms based on preliminary data on the association between behavioral data on motor features of ASD and ASD symptom severity.

## Methods

### Study sample

In total, we examined 82 subjects, thirty-seven adults with ASD without intellectual impairment (age mean ± standard deviation 36.9 ± 1.7 years, 18 females) and forty-five healthy controls (age 33.0 ± 1.3 years, 24 females), matched for age and sex. Included were individuals with IQ over 85 to ensure the comprehension and correct execution of the instructions. Current anticonvulsant medication was an exclusion criterion to rule out potentially confounding neurological side effects. Also, comorbid neurological disorders and age over 65 years were excluded to avoid possible confounding age-related neurodegeneration. Additionally, a history of any psychiatric disorder as well as an Autism Spectrum Quotient (AQ)^30^—a questionnaire on autistic traits—above 32 points in HC led to exclusion.

All participants of the ASD group were diagnosed in the specialized outpatient clinic for ASD at Charité—Universitätsmedizin Berlin, Germany. The diagnostic process comprised gold-standard instruments: the Autism Diagnostic Observation Schedule module 4^31^, German version^32^, clinical semi structured interviews based on the Diagnostic and Statistical Manual of Mental Disorders, 4th edition^33^ ASD criteria, and, if a parent was available (*n* = 21), the Autism Diagnostic Interview—Revised (ADI-R)^34^, German^35^. If a parent was not available, the patient was asked to illustrate sufficient examples of autistic symptoms which had already been present in childhood. The diagnosis of ASD was established by expert consensus while considering clinical interviews and scale assessments. Experienced psychologists and psychiatrists performed the ADOS-2 interview and other interviews, e.g. structured clinical interview I and II of DSM-IV, to examine for other psychiatric disorders according to DSM-IV^36^. Additionally, AQ was assessed in individuals with ASD as well.

HC were recruited through databases of the psychological experimental server from the Humboldt University Berlin, through printed and online bulletin boards at various Charité sites, and through online posts in social media.

### Ethics

The study was approved by the local ethics committee of the Charité Berlin (EA1/392/16) and was conducted in accordance with the Declaration of Helsinki. All participants gave written informed consent before entering the study.

### Clinical assessment

All participants filled in a series of questionnaires, including a demographic questionnaire to obtain participants’ age, sex, level of education, and native language. The structured clinical interview I and II of DSM-IV were performed^36^.Verbal IQ and abnormalities in language understanding were assessed by a German vocabulary test (Wortschatztest (WST))^37^. Quality of Life was assessed with the WHOQOL-BREF measure the following broad domains: physical health, psychological health, social relationships, and environment^38^. The WHOQOL entails an Item of self-reported ”negative feelings (“blue mood”, “despair”, “depression”, “anxiety”). Also, clumsiness and handedness were self-reported in both groups.

### Quantitative motor assessment

Participants performed nine movement tasks that are well established as tests of motor coordination and gross motor functioning which were chosen according to previous descriptions of motor signature in children and adolescents with ASD and have been technically validated in healthy controls^2,15^. A detailed description of the tasks is provided in Table 1.

**Table 1:** Description of the nine Movement Tasks.

Task	Description	Abbreviation	Duration	Repetitions
Postural control—Stance	Standing with parallel closed feet with open eyes, then repeating it with closed eyes	POCO	2 × 20 s	1
Postural control—Tandem stance	Standing in a heel-to-toe position with open eyes, then repeating it with closed eyes	POCO-T	2 × 20 s	3
Single leg stance	Standing on one leg with open eyes	SLS	20 s	1 (each side)
Stepping in place	Walking on the spot in a subjectively comfortable speed	SIP	40 s	1
Paced stepping in place	Walking on the spot in a given pace of 90 steps/min	pSIP	40 s	1
Jumping Jack	Performing 10 jumping jacks continuously	JJ	individual	3
Walk at a subjectively comfortable speed	Walking directly towards the system (4 m) in a subjectively comfortable speed	SCSW	individual	3
Line walk	Walking on an imagined line in a heel-to-toe manner	SLW	individual	3
Finger tapping test	Tapping the index finger and the thumb against each other 10 times as quickly as possible and returning to the original position (wide angle between index finger and thumb)	FTT	individual	3

Filming conditions included light, silhouette-fitting clothing and comfortable footwear of the subjects and an evenly lit environment, free of any distracting objects. Movement tasks were performed following standardized operator instructions. Participants were not allowed to speak during the assessments.

**Figure 1:**
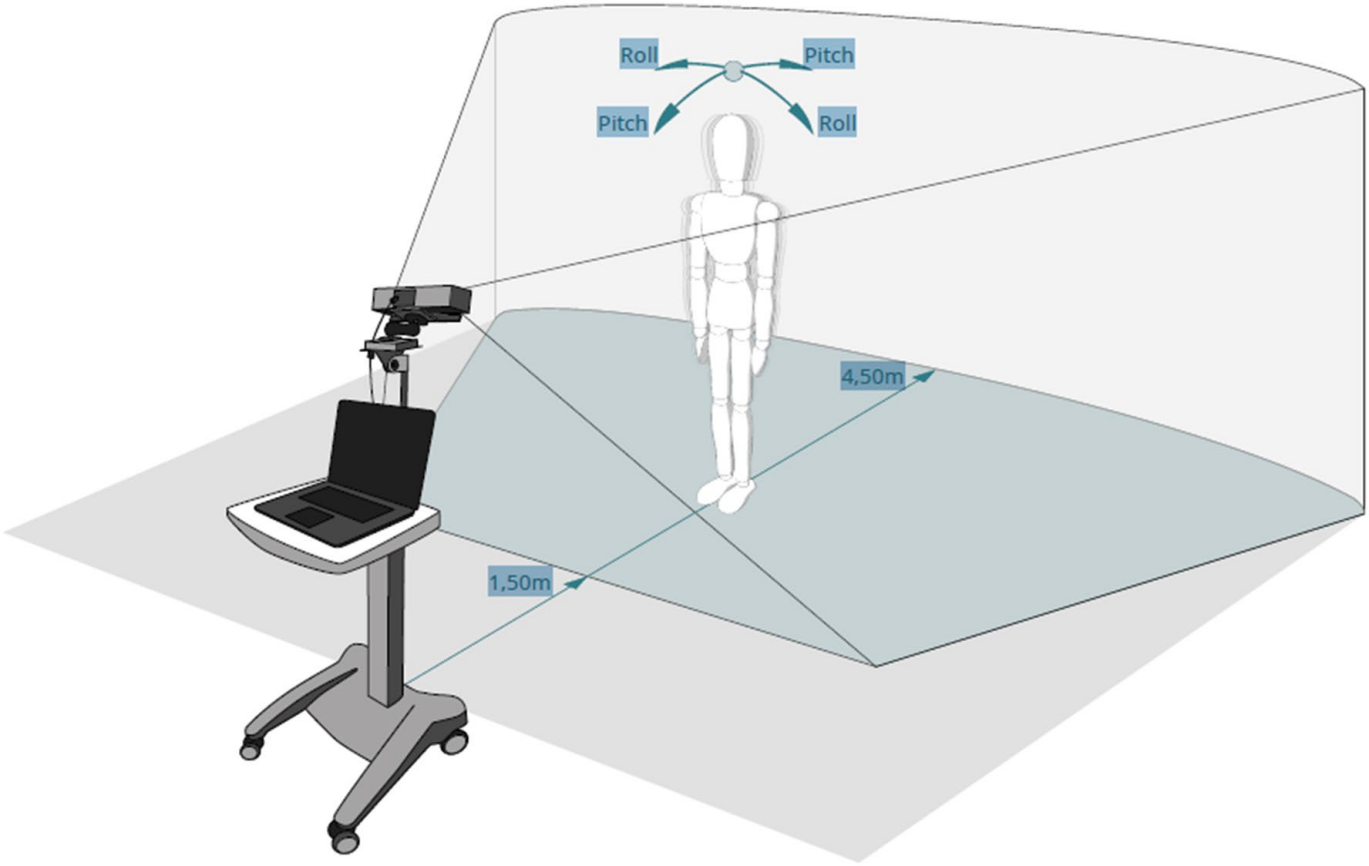
Set-up of quantitative motor assessment by visual-perceptive computing. Rol = anteriorposterior direction, Pitch = mediolateral direction.

Throughout performance of each task, subjects were recorded by a 3D-infrared camera (Microsoft Kinect for Xbox one) at a 30 Hz sampling rate. The sensor was placed on a tripod at 1.4-m height with a vertical angle of − 8°. For all tasks except walking and finger-tapping, participants stood at a distance of 2.5 m from the sensor. To cover full gait cycles, participants started at a distance of 5 m from the sensor, which was slightly outside of the sensor range and then walked straight towards the sensor. The finger-tapping task was performed at a 1.5 m distance from the sensor to enhance the depth resolution of the tracked hands. The video assessment system and the study set-up are illustrated in Fig. 1. Motion data were captured using Motognosis Labs v1.0 software (Motognosis GmbH, Berlin, Germany).

Depth data of the recordings were analyzed to obtain a set of kinematic parameters. These included sway movements of the trunk at hip level during all stance tasks, described as range of 3-dimensional sway movement [°] and mean sway velocity [°/s]. Performance in walking tasks was described as walking speed [m/s] and movement at hip level in the mediolateral direction [cm]. Tests of repetitive movements, e.g. stepping in place, jumping jacks and finger tapping were described by movement frequency. In tests of repeated assessment (e.g. walk at comfortable speed) the mean value was calculated. Arrhythmicity was determined from stepping in place and jumping jack task and asymmetry of stepping described from stepping in place. A description of all 15 outcome parameters is shown in Table 2.

**Table 2:** Description of kinematic parameters to describe performance in the nine movement tasks.

Tasks	Parameters	Unit	Description
POCO, POCO-T, SLS	DR pitch	°	Range of sway movement in anterio-posterior direction
	DR roll	°	Range of sway movement in medio-lateral direction
	MSV 3D	°/s	Mean sway velocity in 3-dimensional space
SLW, SCSW	Speed	m/s	Walking speed
	Mov X	cm	Standard deviation of movements in mediolateral direction
	Mov Y	cm	Standard deviation of movements in vertical direction
SIP, pSIP	Knee Amplitude	m	Antero-posterior range of motion of knees
	Amplitude Asymmetry	%	Logarithmic ratio between the knee amplitudes of the larger side to the smaller side
	Cadence	Steps/min	Steps per minute
	Arrhythmicity	%	Positive ratio between standard deviation and average of the step time for one body side
JJ	Hand Cycle Time	s	Time of Hand movement cycle (from maximal hand distance over minimal hand distance to next maximal hand distance)
	Hand Cycle Arrhythmicity	%	Absolute ratio between standard deviation and average of the hand cycle time
	Feet Cycle Time	s	Time of Feet movement cycle (from maximal feet distance over minimal feet distance to next maximal feet distance)
	Feet Cycle Arrhythmictiy	%	Absolute ratio between standard deviation and average of the feet cycle time
FTT	R, L Frequency	Hz	Main tapping frequency (taps per second)

POCO = postural control stance, POCO-T = postural control tandem stance, SLS = single leg stance, SLW = line walk, SCSW = walk at a subjectively comfortable speed, SIP = stepping in place, pSIP = paced stepping in place, JJ = jumping jack, FTT = finger tapping test, DR = deflection range, MSV 3D = mean sway velocity in 3D, Mov X = movement deviation in mediolateral direction, Mov Y = movement deviation in anteroposterior direction at hip level, R = right, L = left, Hz = Hertz.

### Statistical analysis

Clinical and demographic data and results from questionnaires were compared between adults with ASD without intellectual impairment and HC using a paired-samples t-test. As the main step of analysis quantitative outcomes of virtual-perceptive computing (VPC) were compared between adults with ASD and HC using two-tailed independent t-tests; effect sizes were determined using Cohen’s d with *p* set < 0.05. T-test results were confirmed by using multiple linear regression, controlling for self-reported affective symptoms (item 26) from the WHOQOL. Due to the explorative purpose of this study, *p*-values were not Bonferroni corrected.

For individuals with ASD, the association between quantitative motor outcomes and the ADOS-2 score and AQ was explored by Spearman´s correlation. Statistical analyses were conducted using IBM SPSS Version 22.0.

### Findings

In this pilot study, we examined 82 subjects (ASD: *n* = 37, age mean ± standard deviation 36.9 ± 1.7 years, 18 females; HC: *n* = 45, age 33.0 ± 1.3 years, 24 females). One patient with ASD was excluded from the statistical analysis for not meeting the filming conditions (see data acquisition). The groups did not differ significantly regarding age, sex, verbal IQ, height, handedness, and BMI (Table 3). Individuals with ASD reported significantly more often ‘clumsiness’ in self-reported questionnaires (*p-*value < 0.001) than healthy controls. An overview of clinical and demographic data is displayed in Table 3.

**Table 3:** Clinical and demographic data of participants.

	ASD (*n* = 37)	HC (n = 45)	*p*	Cohen´s-D
Age, years	36.9 (1.7)	33.0 (1.3)	.063	0.363
Female sex, *n* (%)	18 (48.6)	24 (53.3)	.827	0.059
Verbal IQ	111 (2.1)	109 (1.4)	.360	0.236
BMI, kg/cm^2^	25.3 (1.1)	24.0 (.6)	.297	0.188
Height, cm	172.2 (1.9)	173.9 (1.4)	.462	− 0.178
Self-reported clumsiness, n	23 (.45)	01 (.15)	< .001	2.170
ADOS-2 total sum score	9.3 (3.3)			
Autism Spectrum Quotient (AQ)	37.7 (1.0)	14.3 (.9)	< .001	4.045
Non-right-handedness, n (%)	9 (25.0)	7 (15.5)	.275	0.268
Current psychiatric medication	15 (40.5)	0 (0.0)	< .001	1.166
Antidepressants	11 (29.7)			
Self-reported current depressive disorder	7 (18.9)			
WHOQOL sum score	53.00 (23,8)	75.00 (16.6)	< .001	0.258

All measures are presented as means with standard deviation in brackets unless otherwise specified. ADOS-2 = Autism Diagnostic Observation Schedule module 4, AQ = Autism Spectrum Quotient (Baron-Cohen et al., 2001), WHOQOL = The abbreviated version of the WHOQOL, The World Health Organization Quality of Life.

In healthy controls, history of psychiatric disorder or current psychiatric medication were an exclusion criterion. A list of lifetime psychiatric diagnoses and current psychiatric medication in persons with ASD is available in the supplements. Seven individuals with ASD reported having a lifetime diagnosis of a depressive disorder.

In VPC motor assessment, adults with ASD displayed differences in motor task performance compared to HC (see Table 4). During the task ‘walk at a comfortable speed’, adults with ASD walked more slowly than HC. Further, more mediolateral body deviation compared to HCs was observed during walks at comfortable speed and line walk. In stepping in place at self-chosen speed, individuals with ASD chose a lower stepping frequency (cadence) compared to HCs, while no statistically significant differences to HC were observed when the same task was performed with external pacing. Further, a reduced frequency of movement repetitions was seen for gross movements (10 repetitions of Jumping Jacks) as well as fine movements (10 repetitions of finger tapping right hand) in individuals with ASD compared to HCs. Arrhythmicity of JJ feet movement cycles was higher in individuals with ASD than in HC. Of note, arrhythmicity of step time during stepping place task was generally higher than arrhythmicity observed during JJ performance in both, ASD and HC, and group difference did not reach significance.

**Table 4:** Assessment of motor functions in adults with ASD in comparison to HC.

Tasks	Parameter (unit)	HC Mean (SD)	ASD Mean (SD)	Mean Diff	T-Test *p*-value	Cohen´s D
POCO	Eyes Open DR pitch (°)	2.07 (1.09)	2.46 (1.66)	0.39	.213	0.277
	Eyes Open DR roll (°)	1.68 (0.72)	2.27 (1.46)	0.60	**.027**	**0.522**
	Eyes Open MSV 3D (°/s)	0.44 (0.17)	0.59 (0.30)	0.14	**.012**	**0.592**
	Eyes Closed DR pitch (°)	2.39 (1.65)	3.34 (2.68)	0.95	.067	0.426
	Eyes Closed DR roll (°)	3.09 (2.14)	4.72 (4.24)	1.63	**.039**	**0.486**
	Eyes Closed MSV 3D (°/s)	0.72 (0.41)	1.03 (0.69)	0.31	**.019**	**0.552**
SLW	Speed (m/s)	0.29 (0.07)	0.29 (0.12)	0.01	.819	0.050
	Mov X (cm)	1.45 (0.28)	1.66 (0.50)	0.21	**.030**	**0.509**
	Mov Y (cm)	0.97 (0.38)	1.83 (0.94)	0.86	** < .001**	**1.202**
SCSW	Speed (°/s)	1.29 (0.16)	1.06 (0.31)	− 0.24	** < .001**	− **0.962**
	Mov X (cm)	1.68 (0.43)	2.04 (0.64)	0.36	**.004**	**0.659**
	Mov Y (cm)	1.06 (0.28)	1.14 (0.62)	0.08	.445	0.168
SIP	Knee Amplitude (m)	18.1 (5.14)	18.0 (5.87)	− 0.10	.935	− 0.018
	Amplitude Asymmetry (%)	10.08 (9.86)	11.52 (10.85)	1.44	.535	0.139
	Cadence (Steps/min)	93.77 (15.9)	82.76 (17.33)	− 11.01	**.004**	− **0.661**
	Arrhythmicity (%)	9.50 (6.12)	11.35 (7.73)	1.85	.237	0.265
SLS	R DR 3D (°)	5.20 (3.13)	6.27 (3.61)	1.06	.162	0.315
	R MSV 3D (°/s)	0.92 (0.46)	1.02 (0.09)	0.10	.355	0.209
	L DR 3D (°)	4.20 (1.96)	4.38 (0.18)	0.18	.769	0.065
	L MSV 3D (°/s)	0.81 (0.33)	1.05 (0.24)	0.24	**.034**	**0.474**
JJ	Hand Cycle Time (s)	1.18 (0.77)	1.16 (0.42)	− 0.02	.913	− 0.025
	Hand Cycle Arrhymicity (%)	0.10 (0.14)	0.12 (0.18)	0.02	.494	0.153
	Feet Cycle Time (s)	1.02 (0.13)	1.10 (0.25)	0.09	**.035**	**0.466**
	Feet Cycle Arrhymicity (%)	0.05 (0.05)	0.10 (0.12)	0.06	**.009**	**0.626**
pSIP	Knee Amplitude (m)	17.4 (4.67)	17.4 (5.87)	0.00	.981	-0.005
	Amplitude Asymmetry (%)	13.01 (9.40)	12.21 (10.68)	− 0.79	.725	− 0.079
	Cadence (Steps/min)	89.08 (5.45)	87.43 (6.90)	− 1.65	.236	− 0.265
	Arrhythmicity (%)	9.86 (4.32)	9.36 (4.87)	− 0.50	.629	− 0.108
POCO-T	Eyes Open DR 3D (°)	2.34 (1.33)	2.72 (1.77)	0.38	.277	0.243
	Eyes Open MSV 3D (°/s)	0.50 (0.21)	0.67 (0.37)	0.17	**.019**	**0.552**
	Eyes Closed DR 3D (°)	2.83 (2.21)	4.71 (3.87)	1.89	**.011**	**0.599**
	Eyes Closed MSV 3D (°/s)	0.85 (0.53)	1.24 (0.90)	0.39	**.026**	**0.521**
FTT	R Frequency (Hz)	2.08 (0.43)	1.81 (0.53)	− 0.28	**.016**	− 0.571
	L Frequency (Hz)	2.08 (0.57)	1.86 (0.68)	− 0.22	.123	− 0.364

HC = healthy controls, ASD = autism spectrum disorder, SD = standard deviation, Diff = difference, Sem = standard error of measurement, DR 3D = deflection range in 3D, MSV 3D = mean sway velocity in 3D, Mov X = movement deviation in mediolateral direction, Mov Y = movement deviation in anteroposterior direction at hip level, R = right, L = left, Hz = Hertz, POCO = postural control stance, POCO-T = postural control tandem stance, SLS = single leg stance, SLW = line walk, SCSW = walk at a subjectively comfortable speed, SIP = stepping in place, pSIP = paced stepping in place, JJ = jumping jack, FTT = finger tapping test. For description of movement tasks see Table 1. Bold values show *p*-value smaller than .05.

Results were confirmed by multiple linear regression analysis controlling for reported depressive symptoms in WHOQOL-Bref in both groups. No differences in statistically significant results were found (see supplementary materials, Table 3).

**Figure 2:**
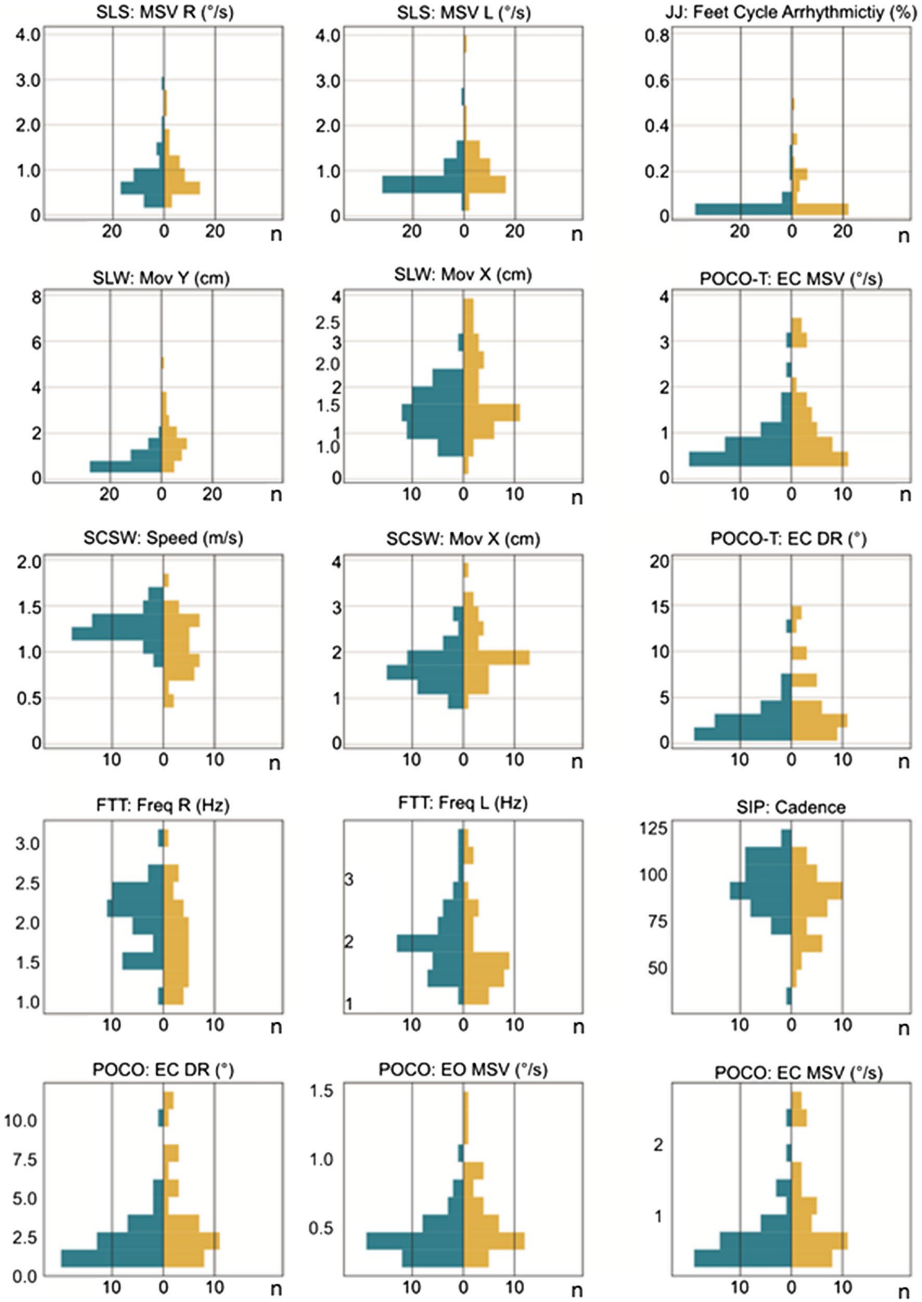
Butterfly histograms for selected quantitative measures from VPC motor assessment (single leg stance, jumping jack arrhythmicity, line walk, stance feet closed, walk in comfortable speed and finger tap test). Performance in ASD/HC are depicted on the right (blue)/left (orange) from midline. HC = healthy controls, ASD = autism spectrum disorder, EC = eyes closed EO = eyes open, DR = deflection range in 3D, MSV = mean sway velocity in 3D, Mov X = movement deviation in mediolateral direction, Mov Y = movement deviation in anteroposterior direction at hip level, R = right, L = left, Hz = Hertz, Freq = frequency, POCO = postural control stance, POCO-T = postural control tandem stance, SLS = single leg stance, SLW = line walk, SCSW = walk at a subjectively comfortable speed, SIP = stepping in place, pSIP = paced stepping in place, JJ = jumping jack, FTT = finger tapping test. For description of movement tasks see Table 1.

A selection of group differences in quantitative measures between ASD and HC, e.g., gait velocity when walking at a comfortable speed, are shown via butterfly histograms in Fig. 2.

When we explored correlations for an association between motor outcomes and severity of ADS symptoms in adults with ASD, this flagged a subset of 9 out of 15 parameters with group differences to HC: body sway velocity in stance eyes open and tandem stance eyes closed, range of 3-dimensional sway at hip level in stance eyes closed, tandem stance eyes closed and single leg stance, walking speed and sway at hip level in mediolateral direction while walking at a subjectively comfortable speed, cadence while stepping in place and finger tapping frequency with the right hand. However, correlations with the ADOS-2 and AQ score severity and were mostly small (*r* < 0.390, see Table 5) and only moderate for the parameter of comfortable walking speed (r = − 0.410 in the correlation analysis with ADOS-2).

**Table 5:** Correlation of quantitative motor outcomes in relation to the ADOS-2 score and AQ score in adults with ASD.

Task	Assessment	Correlation with ADOS-2 Spearman’s Correlation r (2-tailed sig. p)	Correlation with AQ Spearman’s Correlation r (2-tailed sig. p)
POCO	Eyes Open DR pitch	**0.327 (.048)**	0.129 (.455)
	Eyes Open DR roll	0.235 (.162)	0.126 (.464)
	Eyes Open MSV 3D	**0.355 (.031)**	0.177 (.303)
	Eyes Closed DR pitch	0.142 (.401)	0.321 (.057)
	Eyes Closed DR roll	0.220 (.190)	0.255 (.133)
	Eyes Closed MSV 3D	0.159 (.347)	**0.343 (.041)**
SLW	Speed	− 0.049 (.775)	− 0.026 (.879)
	Mov X	0.238 (.156)	− 0.142 (.408)
	Mov Y	-0.019 (.910)	− 0.025 (.887)
SCSW	Speed	**− 0.410 (.012)**	− **0.366 (.028)**
	Mov X	0.319 (.054)	0.281 (.097)
	Mov Y	0.093 (.584)	− 0.177 (.302)
SIP	Knee Amplitude	0.082 (.630)	0.050 (.772)
	Amplitude Asymmetry	0.224 (.182)	0.127 (.461)
	Cadence	**− 0.390 (.017)**	0.139 (.418)
	Arrhythmicity	0.016 (.925)	− 0.074 (.667)
SLS	R DR 3D	0.215 (.201)	0.150 (.384)
	R MSV 3D	0.073 (.666)	0.189 (.271)
	L DR 3D	0.197 (.243)	− 0.127 (.460)
	L MSV 3D	0.122 (.473)	− 0.136 (.428)
JJ	Hand Cycle Time	0.201 (.233)	0.097 (.573)
	Hand Cycle Arrhymicity	0.296 (.075)	− 0.103 (.550)
	Feet Cycle Time	0.120 (.481)	0.070 (.684)
	Feet Cycle Arrhymicity	0.269 (.108)	0.016 (.924)
pSIP	Knee Amplitude	0.073 (.668)	− 0.013 (.939)
	Amplitude Asymmetry	0.034 (.842)	0.141 (.411)
	Cadence	− 0.248 (.139)	0.203 (.235)
	Arrhythmicity	**-0.328 (.048)**	0.179 (.296)
POCO-T	Eyes Open DR 3D	0.312 (.060)	0.167 (.330)
	Eyes Open MSV 3D	**0.359 (.029)**	0.152 (.375)
	Eyes Closed DR 3D	0.204 (.227)	0.288 (.089)
	Eyes Closed MSV 3D	0.140 (.409)	**0.331 (.048)**
FTT	R Frequency	**0.366 (.033)**	− 0.122 (.498)
	L Frequency	0.278 (.117)	− 0.283 (.116)

ADOS-2 score = Autism Diagnostic Observation Schedule module 4 total score, AQ = Autism Spectrum Quotient, ASD = autism spectrum disorder, DR 3D = deflection range in 3D, MSV 3D = mean sway velocity in 3D, Mov X = movement deviation in mediolateral direction, Mov Y = movement deviation in anteroposterior direction at hip level, R = right, L = left, POCO = postural control stance, POCO-T = postural control tandem stance, SLS = single leg stance, SLW = line walk, SCSW = walk at a subjectively comfortable speed, SIP = stepping in place, pSIP = paced stepping in place, JJ = jumping jack, FTT = finger tapping test. For description of movement tasks see Table 1.

Significant values are in bold.

## Discussion

Adults with ASD without intellectual impairment showed significantly slower movements as seen in reduced gait velocity, reduced cadence when stepping in place and reduced tapping frequency of the right hand. Further, repetition of gross leg movements showed higher arrhythmicity compared to performance in healthy controls. Another feature of motor performance was related to balance control, as indicated by increased sway velocity during different stance conditions and by increased mediolateral body movements during walking tasks. In sum, our results suggest that adults with ASD without intellectual impairment display a specific motor signature compared to HC that comprises aspects of movement timing and postural control. Furthermore, some motor features showed small to moderate correlation with the severity of autistic symptoms.

Similar to preliminary findings reported for children and adolescents with ASD, our data support a specific motor signature in adult ASD without intellectual impairment^24^. Adults with ASD without intellectual impairment showed slower movements as seen in reduced gait velocity and cadence, while a reduced gait velocity is not specific for ASD and has been described in other psychiatric disorders, e.g., depression^39^. Comparable speed during paced SIP and reduced speed when asked to walk at a comfortable speed in our findings suggests that adults with ASD are essentially able to walk faster, but feel more comfortable walking at a reduced gait velocity. In a previous study, children with ASD showed an increased stride width and a decreased stride length, which would allow a wider base of support while walking^18,40^. This, and the reduced gait velocity and cadence may hint to a tendency in individuals with ASD to enhance their stability while walking. In a study by Nayate and colleagues, children with ASD, when asked to walk at a preferred speed, then at faster and at a slower rate, widened their base of support at a faster walking rate^41^. Stride length and cadence were however not assessed during gait tasks in our study.

Increased postural instability in subjects with ASD compared to HC is also seen in increased sway movements in mediolateral direction during walking tasks. This is in line with previous findings of postural instability during gait in adults with ASD without intellectual impairment^25^. Increased sway in mediolateral direction during stance tasks may hint to an increased postural instability^42^; however, sway velocity during stance tasks were not assessed separately for mediolateral and anteroposterior direction in our study.

Markou and colleagues have reported a higher prevalence of atypical handedness (mixed handedness, ambidexterity, and left-handedness) in individuals with ASD in a meta-analysis and have interpreted this as increased lateralization in persons with ASD^43^. In our cohort, there was a tendency towards non-right-handedness among individuals with ASD compared to HC, however, this trend not reach statistical significance and only relied on self-report. Since the finger tapping task may indicate manual dexterity, our results of a reduced finger tapping frequency when using the right hand, but not the left hand in persons with ASD are in line with previous studies on atypical handedness^44^. Further, the assessment of arrhythmicity and the dynamics of the movement amplitude might be added in future studies to assess the possibility of cerebral and/or cerebellar affectation.

Impaired vestibular control plus fine and gross motor abnormalities have been liked to cerebellar abnormalities in studies investigating adolescents with ASD^42,45,46^. Specifically, a reduced number of Purkinje cells in the cerebellum, a hypotrophy of cerebellar hemispheres and subregions of the vermis and lastly, an impaired connectivity between basal ganglia and the cerebellum were described. Insight into the neurobiological background of motor function differences in ASD may be helpful to better understand the pathophysiology of ASD.

Another important finding of this study is the positive correlation between some motor functions disparities and severity of autism (ADOS-2 module 4 score and AQ score) in adults with ASD without intellectual impairment. Social motor synchronization, the automatic coordination of one´s body to the social environment, is essential for interacting with others and is negatively associated with autism severity^47^. Motor empathy is defined as the tendency to automatically imitate and synchronize facial expressions and movements of another person^48^. Imitating the movements of others is proposed to lead to prosocial behavior in interpersonal interactions and also lead to a better introspection of one´s cognitive processing styles and self-regulatory abilities^49^. In our findings, gait speed when walking in a comfortable speed was comparable in adults with ASD without intellectual impairment despite varying body dimensions. Motor signs in individuals with ASD, such as lower stepping frequency (cadence) in stepping in place at self-chosen speed, while no differences to HC were observed when the same task was performed with external pacing, may lead to reduced social synchronization.

One strength of this study is the quantitative assessment of motor function signature in persons with ASD using a 3D-infrared sensor camera (Microsoft Kinect®^)^ and visual-perceptive computing. Further assessments may implement more various kinetic parameters, such as cadence and stride length during walking tasks. Future studies should extent analysis to individuals with intellectual impairment. Also, this method should be replicated to explore subgroups within persons with ASD, e.g. persons with ASD with varying motor signature, persons with ASD with or without intellectual impairment.

## Limitation

Due to restricted sample size, sex differences were not analyzed in our sample. However, sex distribution was statistically not significantly different between the two observed groups. Considering the high representation of male participants in previous studies on autism spectrum disorder in clinical settings^50^, the not significantly different ratio of female to male participants in our study acts rather as a strength of this study.

It is important to mention that psychiatric comorbidities in individuals with ASD were not an exclusion criterion in this study. Psychiatric diagnoses cannot be fully ruled out as confounding factors that may account for some of the significant differences of the ASD vs the HC group. However, the findings did not alter when controlling for depressive symptoms (item 26 of WHOQOL) as shown in the supplementary materials (Table 3). In addition, the psychiatric comorbidities of the ASD group in this study are representative of the general ASD population without intellectual impairment and, therefore, promote a naturalistic character of this study^28^. Since the operator was trained to read aloud standardized instructions during motor assessments and the participants were not allowed to speak, the bias in instruction can be assumed to be marginal.

Also, the association between motor function and ASD symptom severity should be carefully interpreted due to the explorative nature of this study. Most of the correlations between motor parameters ADOS-2 score and AQ score severity were small (*r* < 0.390) and only moderate for the parameter of comfortable walking speed in the correlation analysis between motor signature and the ADOS-2 score.

VPC appears to be a feasible instrument for the detection of multiple motor function abnormalities in persons with ASD. Future studies should aim to establish the specificity of ASD motor signature by comparison to findings in other psychiatric or neurodegenerative disorders. Studies exploring neuroanatomical and neurofunctional correlates of our findings would help to form hypotheses on their origin. Furthermore, future studies are warranted to account for heterogeneity in ASD phenomenology that includes, but is not restricted to motor functions^51,52^. To implement VPC as a diagnostic tool, a standardized motor score via video analysis should be developed and its validity tested on additional ASD cohorts and clinical control groups. Lastly, since the ASD symptom severity correlated to some motor dysfunctions in individuals with ASD, our results may serve as a starting point to explore therapeutic potential, for instance as biofeedback therapy based on video analysis of motor behaviour. First data show e.g., that real-time biofeedback using Kinect for physical rehabilitation in patients with total knee replacement therapy led to an improvement in gait and quality of life^53^.

## Conclusion

Our results indicate that adults with ASD without intellectual impairment show a specific motor signature compared to HC related to movement timing and postural control and possibly manual dexterity. The data specify knowledge from clinically descriptions of motor functions in ASD. 3D-video analysis appears to be a feasible instrument for a simple and quantitative assessment of multiple motor functions in patients with ASD with potential for use in research and clinical context.

## Supplementary Information

Supplementary Information 1.

Supplementary Information 2.

Supplementary Information 3.

## Data Availability

The data will be provided upon request. To request data, please contact the corresponding author, An Bin Cho (an-bin.cho@charite.de).
