# Supplementary material for: Motor signature of autism spectrum disorder in adults without intellectual impairment
Source: Sci Rep. 2022 May 10;12:7670. doi: 10.1038/s41598-022-10760-5 (PMC9090847; doi:10.1038/s41598-022-10760-5)
Supplement: Supplementary file 1 — Supplementary Information 1. [file 41598_2022_10760_MOESM1_ESM.docx]

**Supplement 1. List of current psychiatric medication in the ASD group**

| **Current medication** | **n** |
| --- | --- |
| **Antidepressants** | 9 |
| Citalopram | 2 |
| Venlafaxin | 2 |
| Bupropion | 2 |
| Doxepin | 1 |
| Agomelatin | 2 |
| **Antipsychotics** | 2 |
| Quetiapin | 1 |
| Perazin | 1 |
| **Psychostimulants** | 4 |
| Methylphenidat | 2 |
| Amphetamin | 2 |
| **Anxiolytics** | 1 |
| Opipramol | 1 |
| Pregabalin | 1 |
