# Supplementary material for: Motor signature of autism spectrum disorder in adults without intellectual impairment
Source: Sci Rep. 2022 May 10;12:7670. doi: 10.1038/s41598-022-10760-5 (PMC9090847; doi:10.1038/s41598-022-10760-5)
Supplement: Supplementary file 2 — Supplementary Information 2. [file 41598_2022_10760_MOESM2_ESM.docx]

**Supplement 2. List of lifetime psychiatric diagnoses in the ASD group**

| **Lifetime psychiatric diagnoses (ICD-10)** | N |
| --- | --- |
| **Depressive disorder** | 7 |
| F32.- | 2 |
| F32.0 | 1 |
| F32.1 | 1 |
| F33.0 | 2 |
| F33.1 | 1 |
| **Anxiety disorder** | 7 |
| F40.1 | 5 |
| F40.01 | 1 |
| F41.1 | 1 |
| **Obsessive-compulsive disorder** | 2 |
| F42.0 | 1 |
| F42.1 | 1 |
| **Posttraumatic stress disorder** | 2 |
| F43.1 | 2 |
| **Attention-deficit hyperactivity disorder** | 1 |
| F90.0 | 1 |
