# Supplementary material for: Motor signature of autism spectrum disorder in adults without intellectual impairment
Source: Sci Rep. 2022 May 10;12:7670. doi: 10.1038/s41598-022-10760-5 (PMC9090847; doi:10.1038/s41598-022-10760-5)
Supplement: Supplementary file 3 — Supplementary Information 3. [file 41598_2022_10760_MOESM3_ESM.docx]

Supplement 3. **Assessment of motor functions in adults with ASD in comparison to HC while controlling for depressive symptoms (Item 26) according to WHOQOL**

|  |  | **F-statistics**  **p-value** | **R-square** | **Group**  **p-Value** | **Group**  **beta** | **ASD CI** | **Item 26**  **p-Value** | **Item 26 Coeff** | **Item 26 CI** |
| --- | --- | --- | --- | --- | --- | --- | --- | --- | --- |
| *POCO* | **Eyes Open DR pitch** | 0.171 | 0.045 | 0.220 | -0.380 | (-0.99; 0.23) | 0.160 | 0.010 | (-0.01; 0.03) |
|  | **Eyes Open DR roll** | **0.041** | 0.080 | **0.021** | -0.600 | (-1.10; -0.09) | 0.324 | 0.010 | (-0.01; 0.02) |
|  | **Eyes Open MSV 3D** | **0.015** | 0.103 | **0.009** | -0.140 | (-0.25; -0.04) | 0.216 | 0.000 | (-0.00; 0.01) |
|  | **Eyes Closed DR pitch** | 0.073 | 0.066 | 0.059 | -0.940 | (-1.91; 0.04) | 0.202 | 0.020 | (-0.01; 0.05) |
|  | **Eyes Closed DR roll** | 0.061 | 0.070 | **0.031** | -1.620 | (-3.09; -0.15) | 0.350 | 0.020 | (-0.02; 0.07) |
|  | **Eyes Closed MSV 3D** | **0.024** | 0.092 | **0.015** | -0.310 | (-0.56; -0.06) | 0.225 | 0.000 | (-0.00; 0.01) |
| *SLW* | **Speed** | 0.646 | 0.011 | 0.832 | 0.000 | (-0.05; 0.04) | 0.366 | 0.000 | (-0.00; 0.00) |
|  | **Mov X** | 0.069 | 0.067 | **0.024** | -0.210 | (-0.39; -0.03) | 0.644 | 0.000 | (-0.00; 0.01) |
|  | **Mov Y** | **0.000** | 0.289 | **0.000** | -0.860 | (-1.17; -0.55) | 0.340 | 0.000 | (-0.01; 0.01) |
| *SCSW* | **Speed** | **0.000** | 0.241 | **0.000** | 0.240 | (0.13; 0.34) | **0.040** | 0.000 | (0.00; 0.01) |
|  | **Mov X** | **0.012** | 0.109 | **0.004** | -0.360 | (-0.60; -0.12) | 0.455 | 0.000 | (-0.00; 0.01) |
|  | **Mov Y** | 0.199 | 0.041 | 0.424 | -0.080 | (-0.29; 0.12) | 0.105 | -0.010 | (-0.01; 0.00) |
| *SIP* | **Knee Amplitude** | 0.821 | 0.005 | 0.927 | 0.000 | (-0.02; 0.03) | 0.534 | 0.000 | (-0.00; 0.00) |
|  | **Amplitude Asymmetry** | 0.643 | 0.011 | 0.545 | -1.410 | (-6.04; 3.21) | 0.481 | 0.050 | (-0.09; 0.20) |
|  | **Cadence** | **0.004** | 0.131 | **0.004** | 10.900 | (3.57; 18.23) | 0.107 | -0.190 | (-0.42; 0.04) |
|  | **Arrhythmicity** | 0.496 | 0.018 | 0.240 | -1.850 | (-4.95; 1.26) | 0.919 | 0.000 | (-0.09; 0.10) |
| *SLS* | **R DR 3D** | 0.369 | 0.026 | 0.163 | -1.070 | (-2.58; 0.44) | 0.828 | -0.010 | (-0.05; 0.04) |
|  | **R MSV 3D** | 0.581 | 0.014 | 0.362 | -0.090 | (-0.30; 0.11) | 0.629 | 0.000 | (-0.00; 0.01) |
|  | **L DR 3D** | 0.566 | 0.015 | 0.783 | -0.170 | (-1.36; 1.03) | 0.307 | 0.020 | (-0.02; 0.06) |
|  | **L MSV 3D** | 0.100 | 0.058 | **0.035** | -0.240 | (-0.47; -0.02) | 0.732 | 0.000 | (-0.01; 0.01) |
| *JJ* | **Hand Cycle Time** | 0.918 | 0.002 | 0.908 | 0.020 | (-0.27; 0.31) | 0.690 | 0.000 | (-0.01; 0.01) |
|  | **Hand Cycle Arrhymicity** | 0.792 | 0.006 | 0.497 | -0.020 | (-0.09; 0.05) | 0.972 | 0.000 | (-0.00; 0.00) |
|  | **Feet Cycle Time** | 0.098 | 0.058 | **0.037** | -0.090 | (-0.17; -0.01) | 0.627 | 0.000 | (-0.00; 0.00) |
|  | **Feet Cycle Arrhymicity** | **0.016** | 0.102 | **0.005** | -0.060 | (-0.09; -0.02) | 0.429 | 0.000 | (-0.00; 0.00) |
| *FTT* | **R Frequency** | 0.055 | 0.079 | **0.017** | 0.280 | (0.05; 0.50) | 0.821 | 0.000 | (-0.01; 0.01) |
|  | **L Frequency** | 0.255 | 0.038 | 0.123 | 0.230 | (-0.06; 0.52) | 0.542 | 0.000 | (-0.01; 0.01) |
| *POCO-T* | **Eyes Open DR 3D** | 0.236 | 0.037 | 0.285 | -0.37 | (-1.06; 0.32) | 0.192 | 0.01 | (-0.01; 0.04) |
|  | **Eyes Open MSV 3D** | **0.024** | 0.092 | **0.015** | -0.17 | (-0.30; -0.03) | 0.229 | 0 | (-0.00; 0.01) |
|  | **Eyes Closed DR 3D** | **0.017** | 0.1 | **0.008** | -1.87 | (-3.25; -0.49) | 0.288 | 0.02 | (-0.02; 0.07) |
|  | **Eyes Closed MSV 3D** | **0.034** | 0.084 | **0.021** | -0.38 | (-0.71; -0.06) | 0.233 | 0.01 | (-0.00; 0.02) |
| *pSIP* | **Knee Amplitude** | 0.805 | 0.006 | 0.972 | 0 | (-0.02; 0.02) | 0.512 | 0 | (-0.00; 0.00) |
|  | **Amplitude Asymmetry** | 0.269 | 0.034 | 0.701 | 0.86 | (-3.57; 5.28) | 0.115 | 0.11 | (-0.03; 0.25) |
|  | **Cadence** | 0.475 | 0.019 | 0.237 | 1.66 | (-1.11; 4.43) | 0.762 | 0.01 | (-0.07; 0.10) |
|  | **Amplitude Asymmetry** | 0.585 | 0.014 | 0.618 | 0.51 | (-1.53; 2.56) | 0.360 | 0.03 | (-0.03; 0.09) |

*Note*. HC = healthy controls, ASD = autism spectrum disorder, SD = standard deviation, Diff = difference, Sem = standard error of measurement, DR 3D = deflection range in 3D, MSV 3D = mean sway velocity in 3D, Mov X = movement deviation in mediolateral direction, Mov Y = movement deviation in anteroposterior direction at hip level, R = right, L = left, Hz = Hertz, POCO = postural control stance, POCO-T = postural control tandem stance, SLS = single leg stance, SLW = line walk, SCSW = walk at a subjectively comfortable speed, SIP = stepping in place, pSIP = paced stepping in place, JJ = jumping jack, FTT = finger tapping test. R Square = Coefficient of Determination, CI = confidence interval, Item 26 Coeff = regressison coefficient controlling for Item 26.. For description of movement tasks see Table 1. Bold values show p-value smaller than .05. Unstandardized beta coefficients are reported.
